# Hemerythrins in the microaerophilic bacterium *Campylobacter jejuni* help protect key iron–sulphur cluster enzymes from oxidative damage

**DOI:** 10.1111/1462-2920.12341

**Published:** 2013-12-17

**Authors:** John J Kendall, Angelica M Barrero-Tobon, David R Hendrixson, David J Kelly

**Affiliations:** 1 Department of Molecular Biology and Biotechnology, The University of SheffieldSheffield, UK; 2 Department of Microbiology, University of Texas Southwestern Medical CenterDallas, TX, USA

## Abstract

Microaerophilic bacteria are adapted to low oxygen environments, but the mechanisms by which their growth in air is inhibited are not well understood. The citric acid cycle in the microaerophilic pathogen *Campylobacter jejuni* is potentially vulnerable, as it employs pyruvate and 2-oxoglutarate:acceptor oxidoreductases (Por and Oor), which contain labile (4Fe-4S) centres. Here, we show that both enzymes are rapidly inactivated after exposure of cells to a fully aerobic environment. We investigated the mechanisms that might protect enzyme activity and identify a role for the hemerythrin HerA (Cj0241). A *herA* mutant exhibits an aerobic growth defect and reduced Por and Oor activities after exposure to 21% (v/v) oxygen. Slow anaerobic recovery of these activities after oxygen damage was observed, but at similar rates in both wild-type and *herA* strains, suggesting the role of HerA is to prevent Fe-S cluster damage, rather than promote repair. Another hemerythrin (HerB; Cj1224) also plays a protective role. Purified HerA and HerB exhibited optical absorption, ligand binding and resonance Raman spectra typical of μ-oxo-bridged di-iron containing hemerythrins. We conclude that oxygen lability and poor repair of Por and Oor are major contributors to microaerophily in *C. jejuni*; hemerythrins help prevent enzyme damage microaerobically or during oxygen transients.

## Introduction

The term ‘microaerophile’ refers to those microbes, which although requiring oxygen for growth, are unable to grow at normal atmospheric oxygen tensions; these organisms are adapted to particular environments that contain low oxygen concentrations (Krieg and Hoffman, 1986). *Campylobacter jejuni* is a classical microaerophilic bacterium that conforms to this definition; it has been shown to require small amounts of oxygen for growth, possibly as it relies on a single NrdAB-type ribonucleotide reductase for DNA synthesis (Sellars *et al*., 2002) yet fully aerobic conditions are growth inhibitory. It is commensal in the gastrointestinal tract of poultry, cattle and swine (Nielsen *et al*., 1997) and is the major causative agent of human bacterial gastroenteritis in the western world, commonly contracted through the consumption of contaminated poultry (Janssen *et al*., 2008). Strains of *C. jejuni* are usually cultivated in gas atmospheres containing 3–10% (v/v) oxygen and 5–10% (v/v) carbon dioxide, and are clearly adapted for colonization of the oxygen-limited environment of the intestinal mucosa. However, *C. jejuni* can survive in the external environment, as it must transfer between hosts, so it will also be transiently exposed to higher oxygen concentrations.

Although there are many genera of both free-living and pathogenic microaerophiles, satisfactory molecular explanations for oxygen sensitivity in any of these bacteria are not available. An attractive but simplistic hypothesis would be a deficiency of oxidative stress defence enzymes. All aerobic bacteria risk damage from molecular oxygen, as the stepwise reduction of O_2_ results in the formation of reactive oxygen species such as the superoxide radical (O_2_^−^) and hydrogen peroxide (H_2_O_2_), which must be destroyed. Well-characterized enzymes carry out this function, including superoxide dismutase and a plethora of enzymes for peroxide destruction including catalase, thiol peroxidases, cytochrome *c* peroxidases and rubrerythrins (Mishra and Imlay, 2012). While it is true that some microaerophiles lack one or more of these enzymes (Krieg and Hoffman, 1986), there are many species that possess all of them, including *C. jejuni* (Atack *et al*., 2008; Bingham-Ramos and Hendrixson, 2008; Atack and Kelly, 2009; Pinto *et al*., 2011).

**Figure 1:**
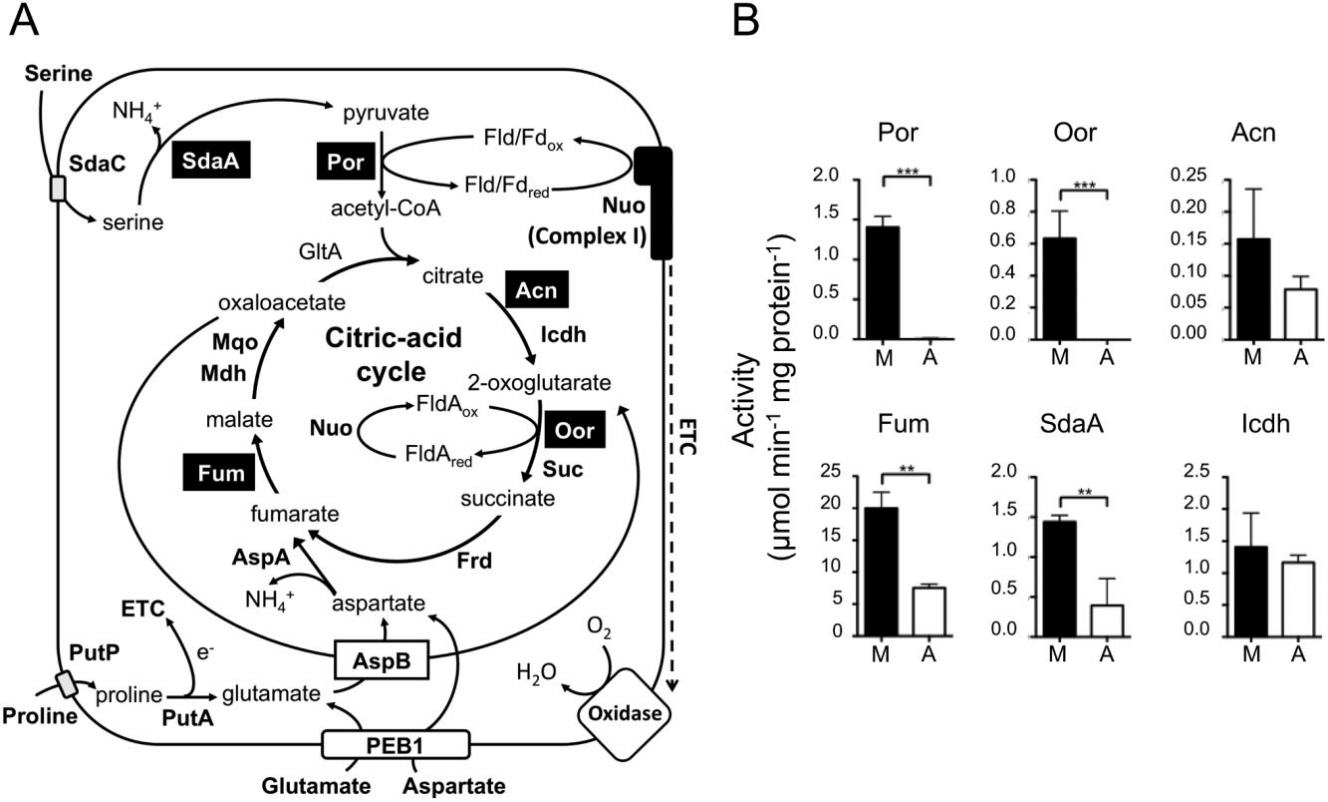
A. Central carbon metabolism of *C. jejuni*. The key enzymes are shown next to the reaction they catalyse. Transporters are shown as rectangles within the cytoplasmic membrane (continuous black line). Fe-S cluster enzymes are highlighted in black text boxes. Key: SdaC, serine transporter; SdaA, serine dehydratase; Por, pyruvate:acceptor oxidoreductase; GltA, citrate synthase; Acn, aconitase; Icdh, isocitrate dehydrogenase; Nuo, flavodoxin/ferredoxin: quinone oxidoreductase; Mqo, malate:quinone oxidoreductase; Mdh, malate dehydrogenase; Oor, 2-oxoglutarate:acceptor oxidoreductase; Suc, succinyl-CoA synthetase; Fum, fumarase; Frd, fumarate reductase [Note: in *C. jejuni* there is no succinate dehydrogenase and the type B fumarate reductase is bi-directional (Guccione *et al*., 2010)]; AspA, aspartase; AspB, glutamate:aspartate aminotransferase; PutP, proline transporter; PutA, proline dehydrogenase. Fld, flavodoxin A; Fd, ferrodoxin; ETC, electron transport chain. B. Specific activities of the Fe-S cluster enzymes Por, Oor, Acn, Fum, SdaA, and the non Fe-S cluster control enzyme Icdh, in cell-free extracts prepared from *C. jejuni* cells incubated for 16 h under microaerobic (M, closed bars) or aerobic (A, open bars) conditions. For aerobic incubations, 50 ml cell suspensions of an OD_600_ 1.0 were placed in 250 ml baffled conical flasks and shaken at 250 r.p.m., as in *Experimental procedures*. The histograms represent the mean activities from three independent cultures, with error bars showing standard deviation. Significant differences are shown as either **P ≤ 0.01 or ***P ≤ 0.001.

One alternative possibility is that microaerophiles, like anaerobes, contain one or more oxygen-sensitive proteins essential for viability, which are directly damaged by high oxygen levels and/or which cannot be repaired at sufficient rates to prevent aerobic growth inhibition. Enzymes that contain iron–sulphur clusters are among the most likely targets for this type of inactivation (Imlay, 2006). In *C. jejuni*, the citric acid cycle (CAC) may be unusually vulnerable to oxidative damage because of the employment of two key iron–sulphur cluster enzymes normally found in obligate anaerobes (Fig. 1A). Firstly, the entry of carbon from pyruvate into the CAC requires its oxidative decarboxylation to acetyl-coenzyme A (acetyl-CoA), which, in most aerobic bacteria, is carried out by the oxygen stable pyruvate dehydrogenase multi-enzyme complex, using NAD as an electron acceptor. However, in many anaerobes, a flavodoxin or ferredoxin-dependent pyruvate:acceptor oxidoreductase (Por) catalyses this essential reaction. Por is an iron–sulphur cluster enzyme containing three (4Fe-4S) clusters and in many anaerobic bacteria is very sensitive to inactivation by molecular oxygen (Pan and Imlay, 2001). Surprisingly for a respiratory bacterium, *C. jejuni* possesses this enzyme (Daucher and Krieg, 1995), as do other *Campylobacter* species and *Helicobacter pylori*, where we previously demonstrated the oxygen lability of the purified enzyme (Hughes *et al*., 1995). Secondly, both *C. jejuni* and *H. pylori* contain a related CAC enzyme, 2-oxoglutarate:acceptor oxidoreductase (Oor) (Hughes *et al*., 1998; Kelly *et al*., 2001) which oxidatively decarboxylates 2-oxoglutarate to succinyl-CoA and which replaces the function of the oxygen-stable 2-oxoglutarate dehydrogenase multi-enzyme complex found in aerobes.

In *C. jejuni*, Oor has been shown to donate electrons to the flavodoxin FldA, which is then re-oxidized by an unusual membrane bound Complex I (Fig. 1A), homologous to the Nuo system of aerobes but lacking the NADH-binding subunits (Weerakoon and Olson, 2008). Electrons from Complex I then reduce the menaquinone pool, which acts as the electron donor for both a low-affinity quinol oxidase and the cytochrome *bc*_1_ complex, which feeds electrons via cytochrome *c* to a very high-affinity *cb*-type cytochrome *c* oxidase (Jackson *et al*., 2007; Kelly, 2008). Therefore, unlike in anaerobes, the 2-oxoacid oxidoreductases in *C. jejuni* act as electron donors to a branched respiratory chain that uses oxygen as terminal electron acceptor. Flavodoxin and (in the absence of an exogenous electron donor) Complex I are known to be essential for viability in *C. jejuni* (Weerakoon and Olson, 2008), underlining the importance of this electron transfer route for microaerobic energy conservation. However, the ready oxidation of low potential flavodoxin by molecular oxygen might present another barrier to fully aerobic growth for *C. jejuni*.

Apart from the (4Fe-4S) dehydratase enzymes aconitase (Acn) and fumarase (Fum) that are both known to be oxidant labile in other bacteria, *C. jejuni* also utilizes an ‘anaerobic-type’ iron–sulphur cluster containing serine dehydratase (SdaA; Fig. 1A), a key enzyme of amino acid catabolism that is necessary for host colonization (Velayudhan *et al*., 2004; Hofreuter *et al*., 2012). When purified, this enzyme is inactivated by molecular oxygen (Velayudhan *et al*., 2004). Although enzymes such as Por, Oor and SdaA have been shown to be oxygen-labile in their purified forms, how (or if) this contributes to the microaerophilic growth phenotype is not clear, as there is no experimental evidence for their oxygen lability in intact cells and no explanation of how they are protected in such a way that allows growth to proceed microaerobically.

In this article, we show that Por and Oor but not the oxygen-stable non-Fe-S enzyme isocitrate dehydrogenase (Icdh) are inactivated after exposure of intact cells of *C. jejuni* to fully aerobic conditions. SdaA, Acn and Fum are also inactivated but to a lesser extent than the 2-oxoacid:acceptor oxidoreductases. Moreover, we have identified a protection system in the form of proteins that show spectral and ligand-binding characteristics typical of hemerythrins. Hemerythrins are μ-oxo-bridged di-iron proteins first identified in certain invertebrates, where they function as oxygen carriers in the absence of haemoglobin (French *et al*., 2008). Genome sequencing has shown that they are also widespread in bacteria and are common in many, but not all, microaerophiles and some anaerobes (French *et al*., 2008). Some bacteriohemerythrins seem to act as oxygen-carrying proteins (Chen *et al*., 2012), while others are found as domains in sensory proteins like diguanylate cyclase (Schaller *et al*., 2012) or DcrH in *Desulfovibrio* *vulgaris* (Xiong *et al*., 2000; Isaza *et al*., 2006). Hemerythrin domains have also been found in a DNase (Padmaja *et al*., 2011) and a P-type ATP solute translocase (Traverso *et al*., 2010) but their roles here are not clear. Our mutant studies in *C. jejuni* strains NCTC 11168 and 81–176 show that at least two of their hemerythrins (Cj0241; HerA and Cj1224; HerB) are important in protecting the activity of Por and Oor from oxygen damage. However, we found no evidence for a role in repair of iron–sulphur clusters after damage has occurred. Under microaerobic (growth) conditions, Por and Oor activity is thus maintained, but fully aerobic conditions for prolonged periods cause irreversible damage. Poor iron–sulphur cluster repair mechanisms may also exacerbate the sensitivity of cells to aerobic growth inhibition and thus also contributes to microaerophily.

## Results

### The iron–sulphur clusters of Por and Oor are highly sensitive to oxygen damage in intact cells of *C. jejuni*

Figure 1B shows the results of enzyme assays in cell-free extracts (CFEs) prepared from wild-type *C. jejuni* NCTC 11168 cells after incubation under standard microaerobic growth conditions or after vigorous shaking in air in baffled shake flasks. In order to ensure that the activities measured in these extracts reflected the effect of the incubation conditions, and not any subsequent effects of oxygen after cell disruption, a glucose oxidase/catalase oxygen scavenging enzyme system was added to the cell samples before they were sonicated and the extracts maintained on ice under anaerobic conditions prior to the assays, which were also carried out under strictly anaerobic conditions. We compared the activities of five key iron–sulphur cluster enzymes which each contain either one (4Fe-4S) cluster (the dehydratases SdaA, Acn and Fum) or three (4Fe-4S) clusters (Por and Oor) with Icdh – a CAC enzyme that lacks iron–sulphur clusters. In the case of the dehydratases, the enzyme activities were all reduced but still detectable after 16 h aerobic incubation compared with their activities in the microaerobic cells at time 0 (Fig. 1B). In contrast, the specific activity of Icdh did not change significantly with this prolonged exposure to atmospheric oxygen (Fig. 1B). However, Por and Oor activities (measured with methyl viologen as electron acceptor) were completely undetectable after 16 h aerobic incubation.

**Figure 2:**
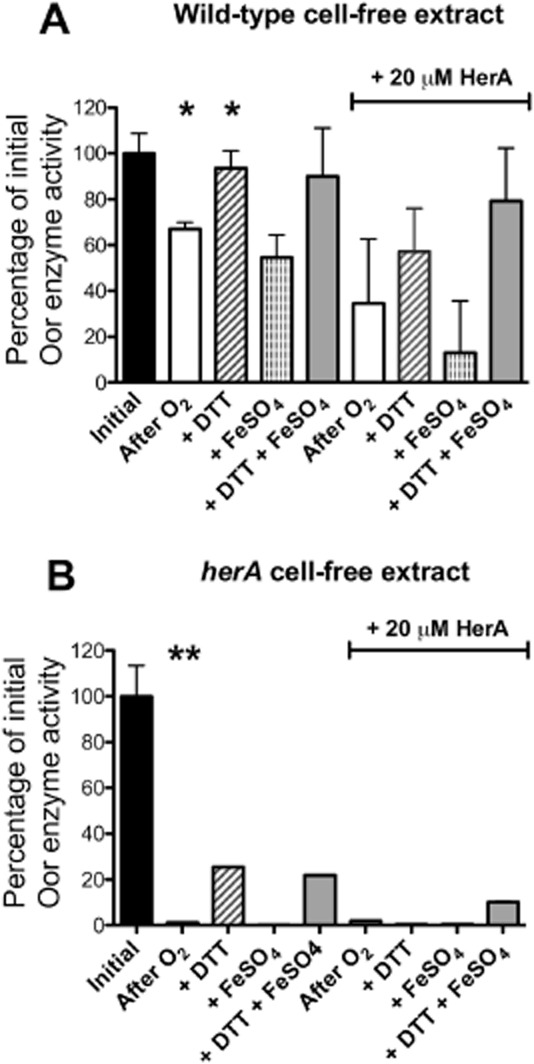
Oxygen damage and *in vitro* recovery of Oor in CFEs prepared from microaerobically grown *C. jejuni* NCTC 11168 wild-type (A) and *herA* mutant (B). CFEs were prepared under anaerobic conditions without the addition of the O_2_ scavenging system and assayed for initial activity of Oor (black bars). The CFE was then exposed to atmospheric oxygen with gentle agitation for 60 min before transfer to anaerobic vessels with or without purified HerA protein (20 μM final concentration). The vessels contained the O_2_ scavenging system to halt aerobic stress and the extracts were assayed again either without any further treatment (after O_2_, open bars) or after the addition of DTT (5 mM; diagonal striped bars) or FeSO_4_ (1 mM; vertical striped bars) or a mixture of both (grey bars). Samples were incubated at 37°C before being assayed for enzyme activity. All values are given as a percentage of the initial pre-stress rates (mean wild-type rate, 1.4 μmol min^−1^ mg protein^−1^; mean *herA* mutant rate, 1.0 μmol min^−1^ mg protein^−1^). Error bars indicate standard deviation of 3 replicate assays. In (A), a significant difference (*; *P* < 0.05) between the initial rate and that after O_2_ exposure was apparent, and between the latter rate and after DTT treatment. There were no significant differences (*P* > 0.05) between the rates in the presence of HerA compared with its absence. In (B) a larger significant decrease (**P < 0.01) in Oor rate occurred after exposure of the *herA* CFE to oxygen, with much poorer recovery by DTT. Rates of treated *herA* CFEs are single measurements.

In order to show that oxygen is specifically damaging the iron–sulphur clusters in these enzymes, the effect of reductant and iron on the reactivation of Oor activity was measured in CFEs. Figure 2A shows that after exposure to aerobic conditions for long enough to cause partial inactivation of Oor, almost complete restoration of activity was possible after anaerobic incubation with dithiothreitol (DTT). Incubation with FeSO_4_ alone had no effect, while incubation with DTT plus FeSO_4_ gave a similar level of recovery to DTT alone (Fig. 2A). This pattern is consistent with oxygen damaging the iron–sulphur cluster(s) in Oor to the (3Fe-4S) stage, which can be reversed by reductant catalysed re-incorporation of the released iron (Djaman *et al*., 2004).

### Kinetics of Por and Oor inactivation correlate with decreases in 2-oxoacid respiration and viability

As Por and Oor are particularly sensitive to inactivation under aerobic conditions in *C. jejuni*, we focused on these enzymes in further work. Repeated attempts to make a deletion mutant in being an essential gene in *C. jejuni*. Construction of an *oorA* mutant has previously been reported (Weerakoon and Olson, 2008) although it is not clear if it has a growth defect, as would be expected.

**Figure 3:**
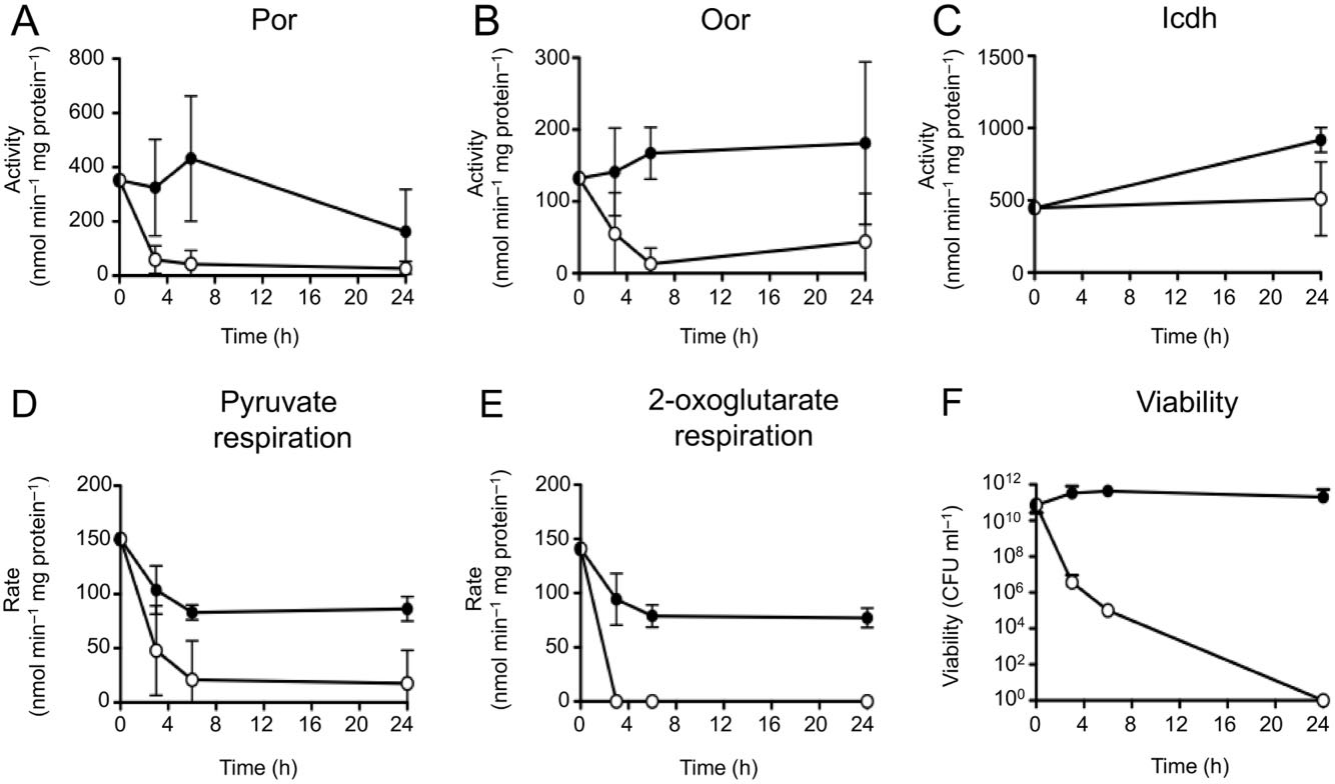
Correlation of the kinetics of oxygen damage to Por and Oor with substrate-dependent respiration rates and cell viability. Cells from early stationary phase microaerobic cultures were resuspended in fresh microaerobically conditioned MHS media to an OD_600_ of 1.0 and incubated under either continued microaerobic conditions (closed circles) or fully aerobic conditions (open circles) conditions for up to 24 h. For aerobic incubation, 500 ml cell suspension was contained in 2.5 l baffled conical flasks and shaken at 250 r.p.m. in a 37°C warm room. The specific activities of Por (A) Oor (B) or Icdh (C) in cell-free extracts prepared from samples taken at the indicated time points are shown. The oxygen-linked respiration rates of pyruvate (D) and 2-oxoglutarate (E) of intact cell samples taken at the same time-points was also determined in an oxygen electrode. The rates were measured over a period of not more than 5 min in the electrode to prevent further enzyme inactivation. In (F), the viable cell count at each time point is shown. The data points represent the mean of three independent experiments, with error bars showing standard deviation.

Wild-type NCTC 11168 cells were resuspended in Mueller-Hinton Serine (MHS) media to OD_600_ 1.0 and incubated either under standard microaerobic growth conditions or shaken vigorously in air in baffled shake flasks as described above and sampled for viable count, enzyme activity (in anaerobically prepared CFEs) and substrate respiration rates over a 24 h period (Fig. 1A). There was a rapid decline in the specific activities of both Por (Fig. 3A) and Oor (Fig. 3B) upon aerobic incubation, such that after 3 h, Por was ∼80% lower than at time zero, and Oor was ∼50% lower, while by 6 h both enzyme activities were almost undetectable. In contrast, in cells incubated microaerobically over the same time period, both Por and Oor activities were maintained at comparable levels to time zero. By 24 h, a decline in Por activity was also apparent in the microaerobically incubated cells. Inactivation of Por and Oor should be reflected in decreased rates of electron transfer from their substrates to oxygen via the respiratory chain. Oxygen electrode measurements showed that both pyruvate- (Fig. 3D) and 2-oxoglutarate (Fig. 3E)-dependent oxygen consumption in intact cells decreased much more markedly during aerobic as compared with microaerobic incubation. For 2-oxoglutarate, respiratory activity became undetectable by 3 h of aerobic incubation. The data in Fig. 3C show that Icdh activity in the aerobically incubated cells did not change over the period of the experiment, and showed a slight increase in microaerobically incubated cells. Microaerobically incubated cells maintained their viability for the duration of the experiment, while aerobic incubation resulted in a dramatic fall in viability (Fig. 3F) consistent with the known oxygen sensitivity of *C. jejuni*.

In separate experiments, we confirmed that the inhibitory effect of oxygen on Por and Oor was due to enzyme inactivation and not due to a shutdown in the expression of the *por* or *oor* genes. In wild-type cells incubated either microaerobically or aerobically for 4.5 h under the same conditions as in Fig. 3, quantitative reverse transcriptase-PCR (qRT-PCR) showed that expression of *por* and *oorA* was not significantly decreased between the two regimes (0.64-fold change, microaerobic to aerobic; *P* = 0.07 and 0.5-fold change, microaerobic to aerobic; *P* = 0.09 respectively). However, in extracts of cells from the same samples, the activity of Por and Oor decreased significantly, from 2.3 to 0.65 μmol min^−1^ mg protein^−1^ (72 ± 1%; *P* = 0.01) and 0.86 to 0.24 μmol min^−1^ mg protein^−1^ (72 ± 1% (*P* = 0.004) respectively.

### Identification and mutagenesis of candidate genes involved in iron–sulphur cluster biosynthesis and repair

In *Escherichia coli* and many other bacteria, at least two genetically distinct systems for iron–sulphur cluster biosynthesis (Isc and Suf) are present (Roche *et al*., 2013). Whereas the genes of the Isc system are constitutive, the *suf* genes are induced by oxidative stress in an OxyR-dependent manner (Jang and Imlay, 2010). The available *C. jejuni* genome sequences show that only some genes of the Isc system are present, with no evidence for a functional Suf system (Supporting Information Table S1). The *iscS (cj0240c)* and *nifU* (*cj0239c*) genes, encoding a cysteine desulphurase and an Fe-S cluster scaffold protein respectively, are the key genes predicted to be involved in the *C. jejuni* Fe-S cluster biosynthesis pathway. The presence of the three-domain NifU, rather than simpler IscU type of scaffold protein in *Epsilonproteobacteria*, was first reported in *H. pylori* (Olson *et al*., 2000). Homologues of the HscA and HscB chaperones, which play a role in the transfer of Fe-S clusters from IscU to apo-enzymes (Roche *et al*., 2013), could not be identified in *C. jejuni*. However, we identified a homologue of the *E. coli* Mrp protein (Cj1606) and a NfuA-like protein (Cj1639), which may also play a role in the later stages of iron–sulphur cluster biogenesis (Supporting Information Table S1; Roche *et al*., 2013). The *cj0239c, cj0240c*, *cj1606* and *cj1639* genes were cloned and insertionally inactivated *in vitro* but repeated attempts to produce null mutants after transformation of *C. jejuni* NCTC 11168 failed, suggesting that they are essential genes.

**Figure 4:**
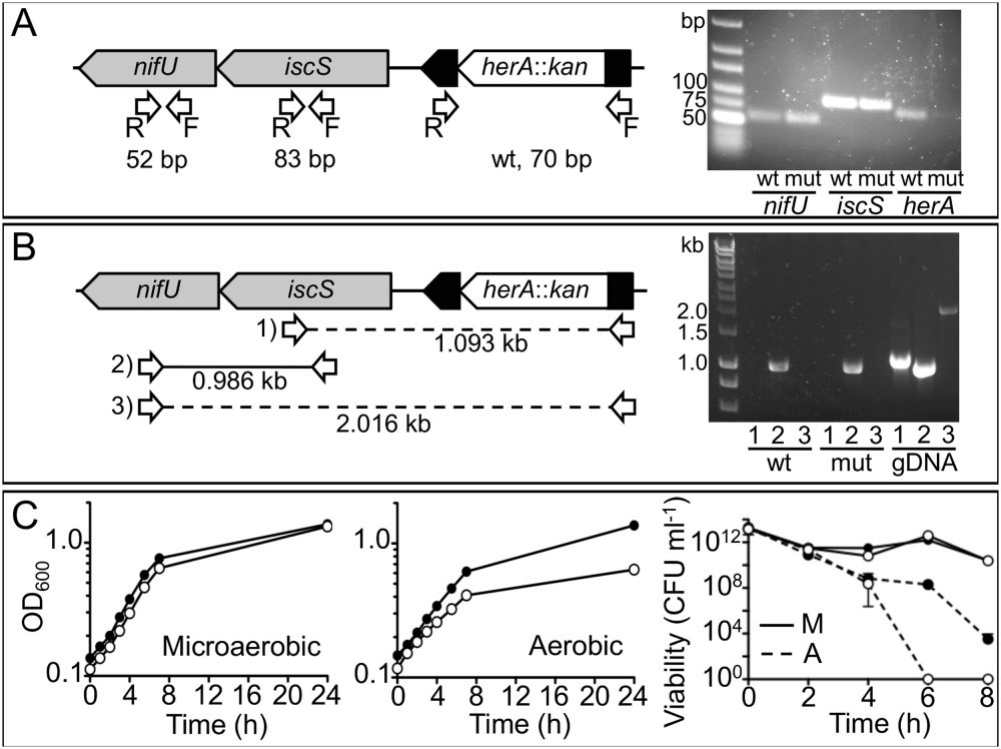
A. Left: Mutagenesis strategy of *herA,* the arrangement of its surrounding gene region and screening for polar effects on expression. A *herA*::*kan* mutant was created by deletion and insertion of a kanamycin resistance cassette to replace most of the coding region of *cj0241c*. *herA* itself is positioned downstream of *cj0243c* (not pictured) and upstream of *iscS* and *nifU*. Arrows indicate RT-PCR primer annealing sites, with expected product sizes shown underneath (bp = base pairs). Right: 3% agarose gel showing results of RT-PCR using cDNA produced from mRNA extracted from wild-type (wt) and *herA*::*kan* (mut) strains, and primer pairs for each gene. B. Left: Further RT-PCR using a combination of primer pairs (numbered). Full lines indicate successful reaction, broken lines indicate unsuccessful. Expected product sizes following RT-PCR are shown underneath each reaction (kb = kilobase pairs). Right: 1% agarose gel showing these reactions with cDNA produced from mRNA extracted from wild-type (wt) and *herA*::*kan* mutant (mut) strains, compared with wild-type genomic DNA as template (gDNA). C. Microaerobic (left panel) and aerobic (middle panel) growth of wild-type (closed circles) and *herA*::*kan* (open circles). For aerobic conditions in this experiment, 50 ml cultures in 250 ml un-baffled conical flasks were shaken at 180 r.p.m. in air (these conditions allowed initial growth of both wild-type and mutant). Right panel: Viability of wild-type (closed circles) and *herA*::*kan* (open circles) during microaerobic incubation (solid line) and aerobic (dashed line) incubation.

The di-iron domain containing protein YtfE has been implicated in iron–sulphur cluster repair in *E. coli* (Justino *et al*., 2007; Vine *et al*., 2010), although its mechanism of action is not understood. There is no homologue of YtfE in *C. jejuni*, but we noted the presence of three complete hemerythrin genes in strain NCTC 11168 (*cj0045c, cj0241c* and *cj1224*; Supporting Information Fig. S1), one or more of which might be candidates encoding functionally analogous enzymes. We initially focused our attention on *cj0241c* (*herA*), as this gene is directly upstream of *iscS* and *nifU*. A null mutant in *herA* was constructed by deleting most of the coding region and replacing it with a kanamycin resistance cassette (Fig. 4A). Despite repeated attempts, we were unable to complement this mutant by integration of the wild-type gene at the *cj0046c* pseudogene locus, which is a standard method used for *C. jejuni* (Shaw *et al*., 2012). Therefore, to ensure the mutation caused no polar effects on the *iscS* and *nifU* genes, mRNA was extracted from wild-type and *herA::kan* strains and subjected to RT-PCR (Fig. 4). Gene specific primers for *iscS* or *nifU* amplified products from both wild-type and *herA* mutant strain cDNA, while *herA* specific primers only amplified a product from the wild-type strain cDNA (Fig. 4A). This suggested no downstream effects on expression of the *iscS and nifU* genes. Moreover, RT-PCR with different combinations of *herA, iscS* and *nifU* forward and reverse primers (Fig. 4B) indicated that *herA* is transcribed independently from *iscS* and *nifU*, which are co-transcribed.

When grown under standard microaerobic conditions, the *herA* mutant did not show any major growth defect (Fig. 4C, left panel), but when grown with slow shaking under aerobic conditions (see *Experimental procedures*), the mutant showed a noticeably reduced growth rate and final cell yield compared with the wild-type (Fig. 4C, middle panel). In cell suspensions incubated with vigorous shaking aerobically, the mutant was also killed more rapidly than the wild-type, while no difference in viability was noted during microaerobic incubations (Fig. 4C, right panel). These phenotypes suggest that HerA plays a significant growth protective role under conditions of oxidative stress.

### HerA has a role in protection of Por and Oor from oxidative damage

**Figure 5:**
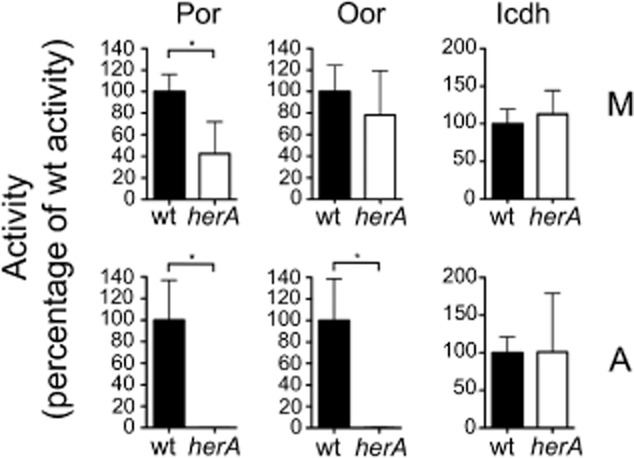
Specific activities of Por, Oor or Icdh in *C. jejuni* wild-type (closed bars) and *herA* mutant (open bars) cell-free extracts prepared from cultures incubated for 16 h under microaerobic (M, top row) or aerobic (A, bottom row) conditions. Aerobic incubation conditions were as in the legend to Fig. 4 (i.e. slow shaking). All activities are expressed as a percentage of the wild-type activity (mean values in nmol min^−1^ mg protein^−1^ for Por M: wt = 370.8, *herA* = 157.8; Por A: wt = 185.5, *herA* = 0.25; Oor M: wt = 491.5, *herA* = 429.5; Oor A: wt = 121.8, *herA* = 0.275; Icdh M: wt = 1390, *herA* = 1568; Icdh A: wt = 1615, *herA* = 1633). The histograms represent the mean activities of three independent cultures, with error bars showing standard deviation. Significant differences are shown as *P ≤ 0.05.

Under standard microaerobic growth conditions in liquid MHS media, the specific activity of Por in early stationary phase (16 h growth) CFEs of the *herA* mutant strain was significantly reduced when compared with the wild-type (Fig. 5, top panels). The mean specific activity of Oor was also lower but greater variation precluded demonstration of statistical significance at *P* < 0.05. However, in anaerobic CFEs of the *herA* mutant subsequently exposed to oxygen (Fig. 2B), Oor was clearly more sensitive to inactivation than in identically treated extracts of wild-type cells, and activity was only poorly restored by DTT treatment (compare Fig. 2A and B).

Under aerobic growth conditions with slow shaking (which allowed growth of both wild-type and mutants strains, see Fig. 4C), neither Por nor Oor activities were detectable by 16 h in the *herA* strain, while they were reduced by ∼50% and ∼70% respectively in the wild-type (Fig. 5, bottom panels). The specific activity of Icdh was not altered in the *herA* mutant under either growth condition compared with wild-type. In separate experiments, qRT-PCR showed that there were no significant differences (*P* > 0.05) between the expression of *por* or *oorA* genes in the wild-type compared with the *herA* mutant under either microaerobic conditions or after shaking in air for 4.5 h.

**Figure 6:**
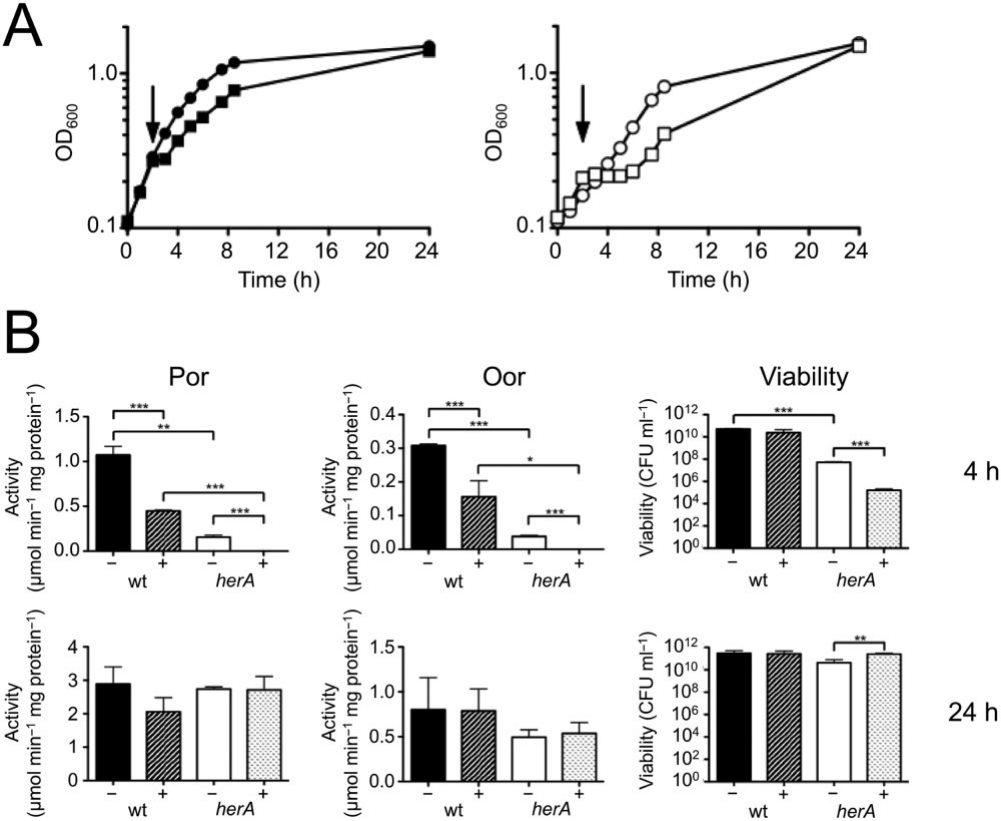
Effect of addition of hydrogen peroxide to *C. jejuni* wild-type and *herA* strains.A. Microaerobic growth of wild-type (left panel) and *herA* mutant (right panel) with (squares) or without (circles) the addition of 0.6 mM H_2_O_2_ during early exponential phase (arrow).B. Top row: Por and Oor enzyme activity in CFE prepared from samples taken during above growth curves at T_4_, and viability of T_4_ sample. Bottom row: Por and Oor enzyme activity in CFE prepared from samples taken during above growth curves at T_24_, and viability of T_24_ sample. Error bars show standard deviation. Significant differences are shown as either *P ≤ 0.05, **P ≤ 0.01 or ***P ≤ 0.001.

To explore the role of HerA further, we exposed cells to hydrogen peroxide, an oxidant that can destroy iron–sulphur clusters directly (Jang and Imlay, 2010). Figure 6 shows that following the addition of 0.6 mM H_2_O_2_ to microaerobically growing *C. jejuni* wild-type and *herA*::*kan* strains in early exponential phase, there is a noticeable lag in growth in both strains; this lasted for about an hour in the wild-type (Fig. 6A, closed squares) but the mutant took ∼4 h to recover fully (Fig. 6A, open squares). Samples were taken at the 4 h time point from which CFE were prepared for Por and Oor assays (Fig. 6B, top row). Interestingly, at this low cell density, even without peroxide treatment, there was a large significant difference in the Por and Oor activities in the mutant strain compared with the wild-type. Addition of H_2_O_2_ had a deleterious effect on the activity of Por and Oor in wild-type cells and reduced the activities to zero in the mutant, making it difficult to assess the precise role of HerA in enzyme protection. However, the viability of the mutant was decreased much more by H_2_O_2_ treatment than in wild-type cells (Fig. 6B). Growth resumed after a period, during which the added peroxide was presumably detoxified by the cells (Fig. 6A), and samples taken at the 24 h time point showed no significant difference in viability or in Por and Oor activity (Fig. 6B, bottom row).

Taken together, the data from these experiments show that in the absence of HerA, Por and Oor enzyme activity is more susceptible to oxidative damage and that HerA also has a role in protection from peroxide stress.

### Evidence that HerA is not involved in the repair of damaged iron–sulphur clusters in Por or Oor

The protective effect of HerA could be due to this protein either preventing the iron–sulphur clusters of Por and Oor being damaged or by promoting their repair after damage has occurred. In order to investigate the latter possibility, the effect of *herA* inactivation on the recovery of enzyme activity after oxidative damage was assessed and the ability of purified HerA to restore activity in CFEs after oxygen exposure was determined.

After exposure of cells to atmospheric oxygen stress for 4.5 h, the cells were transferred to strict anaerobic conditions in the presence of chloramphenicol to prevent *de novo* enzyme synthesis (Djaman *et al*., 2004). The Por and Oor enzyme activities were then measured in CFEs prepared at intervals (Supporting Information Fig. S2). After partial inactivation, the activity of Por increased over a period of a few hours after transfer, but the recovery was slow and incomplete (Supporting Information Fig. S2A). Oor activity showed a more complete recovery (Supporting Information Fig. S2B). However, there was no significant difference (*P* > 0.05) between wild-type and *herA* mutant cells in the rate of enzyme recovery in each case. The data are thus not consistent with a protective effect of HerA associated with oxygen-independent repair. Nevertheless, these experiments establish that *C. jejuni* does have the ability to repair enzyme iron–sulphur clusters to some extent after transient oxidative damage, but the recovery observed is remarkably slow. Anaerobic recovery assays performed after prolonged (16 h) exposure to atmospheric oxygen (Supporting Information Fig. S2C and D) showed that no Por or Oor enzyme activity could be detected even after several hours of anaerobic incubation. Prolonged aerobic exposure clearly damages these enzymes so extensively [i.e. well beyond the (3Fe-4S) stage] that they cannot be repaired at all, consistent with the data in Figs 1 and 3.

**Figure 7:**
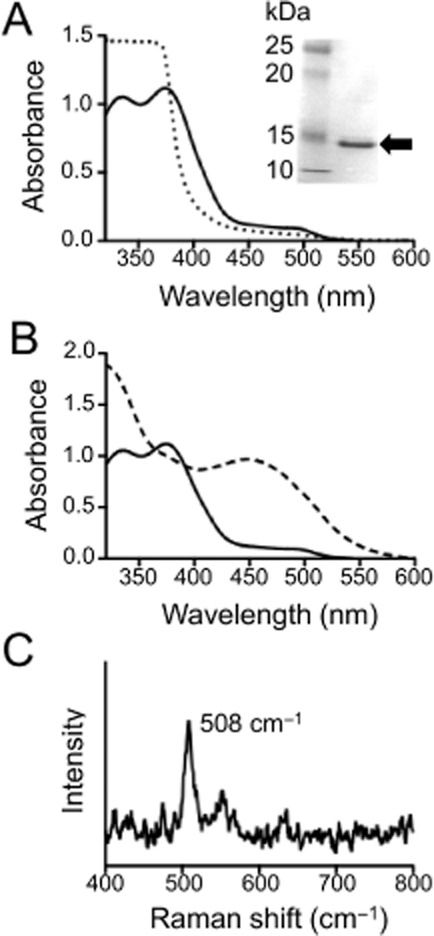
UV-VIS and Raman spectroscopy of purified HerA.A. *Inset*: 12% SDS-PAGE gel showing purified HerA protein band of ∼16 kDa (arrow). *Main figure*: Optical absorption spectra of purified as-isolated HerA (solid line) and HerA reduced with sodium dithionite (dotted line).B. Optical absorption spectra of purified as-isolated HerA (solid line) and HerA after addition of sodium azide (50 mM final concentration; dashed line).C. Resonance Raman spectrum of HerA (1.5 mM protein, in 20 mM sodium phosphate buffer pH 7.4). The feature at 508 cm^−1^ corresponding to the symmetric stretch of the μ-oxo-bridge of the di-iron centre is indicated.

Recombinant his-tagged HerA protein was overproduced in *E. coli* and purified by affinity chromatography (Fig. 7A, *inset*). When the pure protein was added at 20 μM to CFEs of the wild-type strain that had been exposed to oxygen, there was no significant difference (*P* > 0.05) in the Oor activity in comparison with that without added HerA (Fig. 2A). With *herA* CFEs exposed to oxygen, the addition of purified HerA also did not increase the residual Oor activity (Fig. 2B). These data therefore also suggest that HerA is unable to repair damaged iron–sulphur clusters.

### HerA has spectral features typical of di-iron μ-oxo-bridged hemerythrins

Hemerythrins are characterized by a non-heme di-iron core that is not only bridged by the carboxylate side chains of Asp and Glu residues but also is μ-oxo bridged (Fe^III^-O-Fe^III^) in the oxidized form (Kao *et al*., 2008). The iron atoms are liganded by histidine residues, which are all conserved in HerA (Supporting Information Fig. S1). Purified recombinant HerA was greenish-yellow in colour, a characteristic of iron-containing hemerythrins. The ultraviolet-visible (UV-VIS) electronic absorbance spectrum of the as-isolated protein (Fig. 7A) showed two major absorption bands at 336 nm and 374 nm and a weaker feature around 500 nm, which represent ligand-to-metal charge transfer transitions from the μ-oxo bridge to Fe(III) within the di-iron core. These spectral features are diagnostic of the met form of hemerythrins (Kao *et al*., 2008) where a hydroxide is liganded to one of the Fe(III) centres. Reduction of the met protein by dithionite resulted in the bleaching of each of these peaks (Fig. 7A) to form the deoxy form of the protein, where a hydroxide bridges the di-iron binuclear centre, with each iron now in the Fe(II) form. Re-oxidation of the protein by oxygen from the reduced to the peroxy-Fe(III) liganded intermediate can be accompanied by an enhancement of the 500 nm feature, which is diagnostic of the oxy form of hemerythrins (Kao *et al*., 2008) although we did not observe this in our experiments. However, when the as-isolated (met) form of the protein was treated with sodium azide, a characteristic broad absorbance around 450 nm developed (Fig. 7B) because of the formation of an azido-Fe(III)-liganded form. Resonance Raman spectroscopy of the met form of HerA (Fig. 7C) showed a diagnostic feature at 508 cm^−1^, which arises from the symmetric stretch of the μ-oxo-bridge (Kao *et al*., 2008), unequivocally confirming the architecture of the di-iron site.

### The role of other hemerythrins in *C. jejuni*

As indicated above, most strains of *C. jejuni* have a complement of at least three hemerythrin-like proteins. A sequence alignment (Supporting Information Fig. S1) shows that HerA orthologues are the shortest hemerythrins and are essentially identical in strains NCTC 11168, 81116 and 81–176. However, HerB and its orthologues have C-terminal extensions containing some additional conserved histidines as well as CXC and CXXC motifs (Supporting Information Fig. S1) that are characteristic of redox active or metal-binding proteins (Fomenko and Gladyshev, 2003). We cloned and overproduced NCTC 11168 HerB in *E. coli* BL21 (DE3). The optical absorption spectrum of the purified protein was very similar to that of HerA; it was reducible with dithionite and reacted with azide in a similar way (Supporting Information Fig. S3A and B), suggesting that the presence or absence of the C-terminal extension does not affect the hemerythrin-like spectral properties of these proteins.

The Cj0045 protein and its orthologue in strain 81116 also have C-terminal extensions with cysteine motifs. Overproduction of Cj0045 in *E. coli* yielded only insoluble protein, so its properties could not be determined. The closest orthologue of this group in strain 81–176 differs in being smaller and lacking both of these motifs because of the apparent absence of the C-terminal domain (Supporting Information Fig. S1). However, we noted that the intergenic sequence between the stop codon of *cjj81176_0083* and the start codon of *cjj81176_0082* encodes the missing C-terminal domain in a different reading frame (Supporting Information Fig. S1). The *cjj81176_0083* gene has been characterized previously as a **f**lagellar co-**e**xpressed **d**eterminant (FedA) under the control of the alternative sigma-factor σ^28^, and mutant studies have shown that its expression is necessary for chicken colonization and invasion of colonic epithelial cells (Barrero-Tobon and Hendrixson, 2012). 81–176 FedA was successfully overproduced and purified from *E. coli* (Supporting Information Fig. S3C) but, unlike the other hemerythrins studied, it was necessary to supplement the growth medium with ferrous ions in order to obtain a characteristic yellow-green coloured protein; attempts at purification without supplementary iron resulted in the purification of a colourless protein with a UV-VIS spectrum not unlike the dithionite reduced form (Supporting Information Fig. S3C). FedA purified from iron-supplemented cells had similar absorbance features compared with HerA and HerB, although the azido form was less distinct spectrally (Supporting Information Fig. S3D).

**Figure 8:**
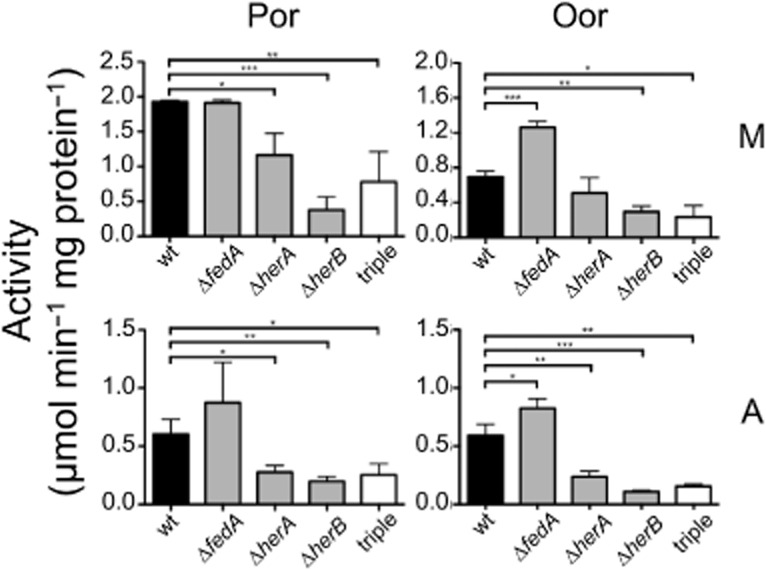
Comparison of the specific activities of Por and Oor in CFEs prepared from *C. jejuni* 81–176 wild-type (black solid bars), the single hemerythrin mutants Δ*fedA*, ΔherA (*0266*) and ΔherB (*1237*) (grey bars), and the triple mutant (open bars) after 20 h microaerobic growth (M, top row) or 16 h microaerobic growth followed by 4 h aerobic stress (50 ml cultures in 250 ml baffled conical flasks, with 250 r.p.m. shaking in air) (A, bottom row). All data points represent the mean average of three biological replicates, with error bars showing standard deviation. Significant differences are shown as either *P ≤ 0.05, **P ≤ 0.01 or ***P ≤ 0.001.

In order to determine if any of these additional hemerythrins also have a role in protection of Por and Oor enzymes, we utilized strain 81–176 where multiple unmarked gene deletions can be made in a facile manner without issues of polar effects (Hendrixson *et al*., 2001). We constructed single deletions in each of the three hemerythrin genes in this strain [*cjj81176_0083* (*fedA*), *cjj81176_0266* (*herA*) and *cjj81176_1237* (*herB*)] and also a triple mutant lacking all of these genes (see *Experimental procedures*). The specific activities of Por and Oor in each strain under microaerobic or aerobic incubation conditions are compared with the wild-type parent strain in Fig. 8. The *fedA* mutant maintained similar activities of Por and Oor after a 4 h aerobic incubation period, while significant reductions in both enzyme activities were found with the Δ*0266* and Δ*1237* single mutants and in the triple mutant, compared with the wild-type. These data show that both the 81–176 HerA and HerB proteins have a role in the protection of Fe-S enzymes, but that FedA does not. A Por recovery assay was performed with the triple mutant but no difference in the rate of recovery of partially oxygen-damaged Por enzyme compared with the wild-type was found after anaerobic incubation (data not shown). This suggests that 81–176 HerA and HerB work by protecting Por from oxygen in a similar way to that seen with NCTC 11168 HerA.

## Discussion

Although a variety of explanations for microaerophilic growth in bacteria have been proposed, there has previously been little experimental evidence for the involvement of specific oxygen-sensitive enzyme targets. This contrasts with the situation for anaerobes, where the physiological consequences of the utilization of oxygen-sensitive enzymes are better understood (Imlay, 2002; 2006,). Here, we provide evidence that the microaerophilic nature of *C. jejuni* is intimately related to its reliance on the flavodoxin- or ferredoxin-dependent Fe-S cluster containing CAC enzymes Por and Oor, which replace the oxygen stable dehydrogenase complexes found in aerobes. Por seems to be an essential enzyme in *C. jejuni* as are flavodoxin and (in the absence of an exogenous electron donor) the Nuo complex (Weerakoon and Olson, 2008). Crucially, both Por and Oor activities are dramatically reduced in *C. jejuni* cells within a few hours of aerobic incubation, and this is accompanied by reductions in both substrate-dependent respiration rates and viability. Our results show that the *C. jejuni* Por and Oor enzymes are far more sensitive to oxygen than Acn, Fum or SdaA, in a similar manner to Por in the anaerobe *Bacteroides thetaiotaomicron* (Pan and Imlay, 2001). The reason for this differential sensitivity is that the Fe-S centre distal to the substrate-binding site has to interact with a large external electron acceptor (flavodoxin/ferredoxin) and is much more solvent exposed than the other clusters in the protein (Imlay, 2006). However, while most Por enzymes from anaerobes have been found to be highly sensitive to molecular oxygen, that from *Desulfovibrio africanus* is remarkably oxygen stable in its as-isolated, purified form (Pieulle *et al*., 1995). This enzyme has a unique C-terminal extension of about 60 amino acids, which can occlude the distal Fe-S cluster in a thioredoxin-dependent, disulphide bond-mediated ‘switch’ between oxidized (oxygen-stable) and reduced (activated) forms (Vita *et al*., 2008; Pieulle *et al*., 2011). Alignments with the *D. africanus* enzyme show that this extension is not present in the *C. jejuni* Por, giving it no intrinsic protection, consistent with the oxygen sensitivity we have demonstrated.

Despite the possession of these very oxygen-sensitive oxidoreductases, microaerophiles like *C. jejuni* are respiratory bacteria that prefer to use oxygen as an electron acceptor, so there must be protective mechanisms that allow such enzymes to operate during normal microaerobic growth. In *E. coli*, the di-iron protein YtfE has been shown to be crucial in maintaining the activities of the Fe-S cluster enzymes Acn and Fum, with the activities of both enzymes not only being reduced in a *ytfE* mutant, but fully recoverable following the addition of purified holo-YtfE protein to CFEs (Justino *et al*., 2007; Vine *et al*., 2010). However, the mechanism of action of YtfE is unknown. *ytfE* mutants are defective in enzyme recovery after oxidative damage, and it has been suggested that YtfE might be an iron donor needed for cluster repair (Py and Barras, 2010) but there is no direct evidence for this. Although it does not have a *ytfE* gene, *C. jejuni* strains possess three genes that are predicted to encode di-iron hemerythrin proteins that we initially hypothesized may play a role in the repair or maintenance of Fe-S clusters in key enzymes of central metabolism. This lead us to examine *herA*, which is found directly upstream of *iscS* and *nifU*, two genes implicated in iron–sulphur cluster biosynthesis. Our results show that mutation of *herA* in *C. jejuni* strain NCTC 11168 renders the bacteria more sensitive to aerobic growth inhibition and results in a negative effect on the specific activities of Por and Oor after oxygen stress. Thus, HerA appears to exert a protective effect on these enzymes *in vivo*. We expected that the mechanism of this protection might be through aiding repair of damaged clusters as in YtfE, but our enzyme recovery experiments did not provide any clear evidence for this. The rate of recovery of Por and Oor activity after partial oxidative damage was found to be slow even in wild-type cells, but the kinetics were not significantly different in the *herA* mutant strain and addition of purified HerA did not aid Oor recovery in CFEs. We therefore conclude that another property of HerA is responsible for its action. One possibility is oxygen sequestration. Hydrophobic residues that form a ‘tunnel’ through which di-oxygen gains access to the iron site (French *et al*., 2008) are conserved in all of the *C. jejuni* hemerythrins (Supporting Information Fig. S1), although further work will be required to demonstrate that they bind oxygen. Nevertheless, oxygen rapidly diffuses across the bacterial cytoplasmic membrane, and so, even if some was bound by the hemerythrins (which would have to be present at micromolar concentrations), continued diffusion would maintain the cytoplasmic oxygen concentration at close to that of the environment. The observation that HerA also seems to protect cells against damage by hydrogen peroxide points to a more complex mechanism that will require further investigation. Interestingly, there is some evidence from a yeast two-hybrid study for a direct interaction of HerA with Por and OorA (Parrish *et al*., 2007), which might explain its mechanism.

An additional hemerythrin, HerB, had very similar optical absorbance and ligand-binding characteristics to HerA, but the role of the cysteine motifs in the C-terminal extension in HerB and related proteins is at present unclear. The data with the single and triple mutants in strain 81–176 suggest that both the HerA- and HerB-like hemerythrins in this strain seem to play a role in Por and Oor protection but that FedA does not. FedA was first characterized in studies aimed at identifying flagellar co-expressed genes and is a colonization factor for strain 81–176 in chickens (Barrero-Tobon and Hendrixson, 2012). Its role may therefore be different from the other hemerythrins studied and may not be related to enzyme protection.

Taking our data together, we propose that under microaerobic conditions, hemerythrins like HerA and HerB help protect oxygen-sensitive enzymes such as Por and Oor. However, there is a limit to this protection, and prolonged aerobic exposure damages these enzymes irreversibly. We argue that such damage strongly contributes to microaerophily in *C. jejuni*. The phylogenetic distribution of bacteriohemerythrins includes some anaerobes and known microaerophiles (French *et al*., 2008), many of which also possess genes for Por and Oor enzymes. Another important factor concerns the effectiveness of repair mechanisms for oxygen-damaged iron–sulphur clusters. In *E. coli*, similar recovery experiments to those performed here show that partially damaged aconitase or fumarase can be fully repaired under anaerobic incubation conditions within minutes of removal of the oxidative stress (Djaman *et al*., 2004). The ability of *C. jejuni* to repair damaged iron–sulphur clusters is seemingly more limited. A complete picture of the mechanism of Fe-S cluster repair processes, even in *E. coli*, is still lacking, but in addition to YtfE, other proteins such as YggX (Gralnick and Downs, 2001) and components of the Suf biogenesis system (Jang and Imlay, 2010) have been suggested to play a role. None of these is present in *C. jejuni*, so it is possible that inefficient repair of enzyme Fe-S clusters may also contribute to the microaerophilic phenotype of this bacterium.

Finally, it might be asked why a respiratory bacterium like *C. jejuni* uses such oxygen-sensitive enzymes as Por and Oor in its CAC. Campylobacters have evolved from anaerobic chemolithoautotrophic *Epsilonproteobacteria* like those that are abundant at hydrothermal vents (Nakagawa *et al*., 2007). These bacteria use the reductive carboxylic acid cycle for CO_2_ fixation (Hügler *et al*., 2005; Nakagawa *et al*., 2007) where the key CO_2_-fixing enzymes are Por and Oor, operating in the reverse direction. As pointed out by Weerakoon and Olson (2008), the ready reversibility of these enzymes probably allowed easy metabolic adaptation from acetyl-CoA and succinyl-CoA carboxylation, to pyruvate and 2-oxoglutarate decarboxylation, respectively, during the evolution of an oxidative CAC in the heterotrophic campylobacters. The legacy, of course, is the oxygen inhibition of these vital reactions, which even with the aid of the sentinel hemerythrins, prevents campylobacters from exploiting fully aerobic environments.

## Experimental procedures

### Media, bacterial strains, growth conditions

*Campylobacter jejuni* NCTC 11168 and 81–176 strains were routinely grown on Columbia agar (Oxoid, Basingstoke, UK) containing 5% (v/v) lysed horse blood and 10 μg ml^−1^ each of amphotericin B and vancomycin at 37°C under microaerobic conditions [10% (v/v) O_2_, 5% (v/v) CO_2_ and 85% (v/v) N_2_] in a MACS growth cabinet (Don Whitley Scientific, Shipley, UK). Selective antibiotics were added at a final concentration of 50 μg ml^−1^ where appropriate. Liquid cultures were grown in Mueller-Hinton broth (Oxoid) additionally containing 20 mM L-serine (MHS) under microaerobic gas conditions as described above, as 50 ml batches in 250 ml conical flasks, or 250 ml batches in 500 ml conical flasks, with continuous shaking at 180 r.p.m. on a KS125 IKA-labortechnic shaker (IKA, Staufen, Germany) in the MACS cabinet. For enzyme assays under fully aerobic incubation conditions (21% v/v O_2_ with atmospheric CO_2_ levels), cells were placed in conical flasks (either 50 ml in 250 ml baffled or un-baffled flasks or 500 ml in 2.5 L baffled flasks as indicated in the appropriate figure legends) with shaking at either 180 or 250 r.p.m. on a Kühner lab-shaker (Infors, Basel, Switzerland) in a 37°C warm room. For *C. jejuni* growth curves, an overnight microaerobic culture was used to inoculate fresh MHS media, supplemented with appropriate antibiotics and equilibrated to microaerobic conditions, to an OD_600_ 0.1, before being incubated either microaerobically or aerobically as above. For growth experiments under peroxide stress, hydrogen peroxide was added to a final concentration of 0.6 mM once the culture had reached exponential phase. Viable counts were determined by plating serial dilutions on MHS agar and incubating microaerobically for 72 h *E. coli* strains DH5α and BL21 (DE3) were routinely grown on solid or in liquid Luria-Bertani (LB) medium (Melford Laboratories, Ipswich, UK) at 37°C, with carbenicillin (100 μg ml^−1^ final concentration) added where appropriate.

### Substrate-dependent respiration rates

Cells were harvested by centrifugation and resuspended in 25 mM phosphate buffer pH 7.4. Respiration rates in response to addition of 5 mM pyruvate or 2-oxoglutarate were measured as described previously (Thomas *et al*., 2011) at 37°C using a Rank Brothers O_2_ Electrode (Rank Brothers Ltd, Cambridge, UK), calibrated with air-saturated 25 mM phosphate buffer pH 7.4, assuming an oxygen saturation of buffer of 220 μM. Whole cell protein was measured using the Lowry assay.

### Enzyme activity assays

For preparation of anaerobic CFEs, cells were harvested by centrifugation and resuspended in 1 ml of N_2_-sparged Tris-HCl buffer (0.1 M, pH 8), to which 0.5 ml of an O_2_-scavenging system [10% (w/v) glucose, 50 μg ml^−1^ glucose oxidase (Sigma, St. Louis, MO, USA), 10 μg ml^−1^ catalase (Sigma)] was then added. The cell suspension was kept on ice and sonicated for three 20 s bursts using a Soniprep 150 (MSE, UK). Following centrifugation, the supernatant (CFE) was decanted into pre-sparged anaerobic vessels. All enzyme assays were performed in a 1 ml total volume in stoppered quartz cuvettes (Hellma, Mullheim, Germany) using a Shimadzu UV-240 recording spectrophotometer (Shimadzu, Kyoto, Japan) with 20–100 μl of CFE per assay. CFE protein concentrations were determined using the Bradford Coomassie blue binding assay (Bio-Rad, Hemel Hempstead, UK).

For Por and Oor assays, the assay mixture [100 mM Tris-HCl pH 8, 0.2 mM CoA-SH (Sigma), 2 mM MgCl_2_.6H_2_O (Sigma), 1 mM methyl viologen (Sigma), 0.1 mM thiamine pyrophosphate (Sigma)] was sparged with N_2_ for 10 min before addition of CFE. The assay was started by the addition of either pyruvate or 2-oxoglutarate to 5 mM final concentration. Rates were calculated using an extinction coefficient of 11.8 mM^−1^ cm^−1^ for methyl viologen at 585 nm. The serine dehydratase (SdaA) assay mixture [50 mM Tris-HCl pH 8, 0.15 mM NADH (Sigma)] was sparged with N_2_ for 5 min before addition of CFE with added purified lactate dehydrogenase as coupling enzyme (10 U; Sigma). The assay was started by the addition of L-serine to 5 mM final concentration and rates measured at 340 nm (NADH extinction coefficient 6.22 mM^−1^ cm^−1^). Aconitase activity was measured at 240 nm (*cis*-aconitate extinction coefficient 3.5 mM^−1^ cm^−1^) by adding CFE and 100 mM sodium isocitrate (Sigma) to 100 mM Tris-HCl pH 8. Fumarase activity was measured at 250 nm (fumarate extinction coefficient 1.48 mM^−1^ cm^−1^) by adding CFE and 100 mM L-malate to 100 mM Tris-HCl pH 8. Isocitrate dehydrogenase activity was measured at 340 nm by adding 5 mM sodium isocitrate to the reaction mixture (50 mM Tris-HCl pH 8, 1 mM NADP, 1 mM MgCl_2_ plus CFE).

### Enzyme recovery assays

Half-litre cultures of *C. jejuni* strains grown under standard microaerobic conditions in MHS media to an OD_600_ ∼1.0 were transferred to 2.5 L baffled flasks and shaken in air (37°C, 250 r.p.m.) for either 4.5 or 16 h. Cells were then harvested by centrifugation and resuspended in an equal volume of fresh MHS media which had been sparged thoroughly with N_2_ to remove any dissolved oxygen and which contained chloramphenicol to inhibit protein synthesis (50 μg ml^−1^ final concentration). The cell suspension was dispensed into 50 ml medical flat bottles that were completely filled and incubated at 37°C for various time periods before CFEs were made as described above.

### DNA manipulation and construction of mutants

Isolation and restriction enzyme digestion of plasmid DNA from *E. coli* was performed as previously described (Sambrook *et al*., 1989). Primers used in this study are listed in Table 1, and PCRs were performed with either Accuzyme (Bioline, London, UK) for cloning or MyTaq (Bioline) for screening. A *cj0241c*::*kan* mutagenic construct was created using isothermal assembly (ISA) cloning (Gibson *et al*., 2009). Two sets of primers (0241 LFF/0241 LFR and 0241 RFF/0241 RFR) were designed to amplify the upstream (left flank) and downstream (right flank) regions surrounding *cj0241c* from strain NCTC 11168 genomic DNA. The LFF and RFR primers contained a 30 bp overlap sequence identical to the sequence immediately upstream (0241 LFF) or downstream (0241 RFR) of the *Hinc*II restriction site of pGEM-3Zf (Promega, Southampton, UK). The LFR and RFF primers each contained 20 bp of the *cj0241c* gene sequence, with the addition of a 30 bp overlap sequence identical to the start (0241 LFR) and end (0241 RFF) sequences of the 1.4 kb kanamycin resistance cassette from plasmid pJMK30 (van Vliet *et al*., 1998), amplified using the Kan F and Kan R primers. The left flank and right flank PCR products, along with the amplified kanamycin resistance cassette, were added in an equimolar ratio with *Hinc*II digested pGEM-3Zf to 15 μl of ISA master mix, consisting of ISA buffer (1 M Tris-HCl pH 7.5, 2 M MgCl_2_, 100 mM dGTP, 100 mM dCTP, 100 mM dTTP, 100 mM dATP, 1 M DTT, 0.25% (w/v) PEG-8000, 100 mM NAD), T5 Exonuclease (Epicentre, Madison, WI, USA), Phusion polymerase (Thermo Scientific, Waltham, MA, USA) and Taq ligase (NEB, Ipswich, MA, USA). The reactions were incubated overnight at 50°C, then purified using the QIAquick® PCR Purification Kit (Qiagen, Limburg, The Netherlands), eluting in 15 μl dH_2_O. The resulting DNA (2 μl) was used to transform competent *E. coli* DH5α, with selection on LB agar containing kanamycin. Colonies were screened by PCR with the flanking primers 0241 LFF and 0241 RFR as well as restriction digest analysis. The resultant plasmid (pGEM0241) contained the extreme flanks of the *cj0241c* gene, interrupted by a kanamycin cassette. Transformation of *C. jejuni* NCTC 11168 by pGEM0241 was performed by electroporation, with transformants selected by growth on plates supplemented with kanamycin. Correct insertion of the resistance cassette was confirmed by colony PCR using the 0241 LFF and 0241 RFR primers.

**Table 1:** Primers used in this study

Primer	Sequence (5′–3′)	Comments
0241_LFF	**GAGCTCGGTACCCGGGGATCCTCTAGAGTC**AACAAGCACTCAAGACAA	**Bolded** sequence homologous to pGEM-3zf directly before *Hinc*II site
0241_LFR	AAGCTGTCAAACATGAGAACCAAGGAGAATGGTTGATATAAGGATATTCT	Underlined sequence homologous to the reverse complement of start of kanamycin cassette
0241_RFF	GAATTGTTTTAGTACCTAGCCAAGGTGTGCAATTATCATCAGTGAAGCTA	Underlined sequence homologous to end of kanamycin cassette
0241_RFR	**AGAATACTCAAGCTTGCATGCCTGCAGGTC**ACTATGTCATCTAGGTCAT	**Bolded** sequence homologous to reverse complement of pGEM-3zf directly after *Hinc*II site
Kan F	ATTCTCCTTGGTTCTCATGTTTGACAGCTTAT	Kan cassette PCR
Kan R	GCACACCTTGGCTAGGTACTAAAACAATTCAT	Kan cassette PCR
iscS FWD	CAAAGGGTGTGGGTGGACTT	RT-PCR screening
iscS REV	ACGACCACCCATGTGTTCTC	RT-PCR screening
0241 FWD	TCAGATGAAGAAGCCTTTATGAGAGA	RT-PCR screening
0241 REV	TTCTGTGAATTCTTGTATGATGGTTGA	RT-PCR screening
0239 FWD	TGCACTGTTCGGTTATGGCT	RT-PCR screening
0239 REV	TAATGCGCTGCTGCTTGTTT	RT-PCR screening
por F	GCTGAGGCCTATGATGGACC	qRT-PCR
por R	GCTCACCTTGCTCTCCTGAG	qRT-PCR
oorA F	GCTATTGGTGCGGCAATGAG	qRT-PCR
oorA R	ATTCCAGGACCGCTACTTGC	qRT-PCR
rpoA F	CGAGCTTGCTTTGATGAGTG	qRT-PCR
rpoA R	AGTTCCCACAGGAAAACCTA	qRT-PCR
0241_OE F	GGGAATTC**CATATG**ACTTATAATGAAAAAAT	**Bold** *Nde*I site
0241_OE R	ATTCCA**CTCGAG**TTTCTTAAATTTTTCTTC	**Bold** *Xho*I site
1224_OE F	GTTAATTC**CATATG**CTTCCAAAATGGGATA	**Bold** *Nde*I site
1224_OE R	ATTCCA**CTCGAG**AGAATATTTTTTGTAAAA	**Bold** *Xho*I site
FedA_OE F	GTTGCTTC**CATATG**GAAGTTAAATG	**Bold** *Nde*I site
FedA_OE R	ATTATA**CTCGAG**AGGTATAATGGATTTCT	**Bold** *Xho*I site

*Campylobacter jejuni* 81–176 mutants were constructed by electroporation following methods described by Hendrixson and colleagues (2001). *Cjj81176_0266* and *Cjj81176_1237* were amplified from *C. jejuni* 81–176 chromosomal DNA with 750 base pairs of upstream and downstream DNA by PCR using primers containing 5′ *Bam*HI restriction sites. Each DNA fragment was then ligated into *Bam*HI-digested pUC19 to create pABT737 (pUC19::*Cjj81176_0266*) and pDRH3256 (pUC19::*Cjj81176_1237*). A SmaI- digested *cat-rpsL* cassette from pDRH265 was ligated into the *Eco*RV site in the coding sequence of each gene to create pABT739 (containing *Cjj81176_0266*::*cat-rpsL*) and pDRH3260 (containing *Cjj81176_1237*::*cat-rpsL*). pABT737 and pDRH3256 were each subjected to PCR-mediated mutagenesis to construct in-frame deletions of the coding sequence of each gene (Makarova *et al*., 2000). For *Cjj81176_0266*, the entire coding sequence was deleted to result in pABT745 (pUC19::Δ*Cjj81176_0266*). For *Cjj81176_1237*, the start codon was fused to codon 156 to result in pABT656 (pUC19::Δ*Cjj81176_1237*). All plasmids were verified by DNA sequencing.

*Campylobacter jejuni* 81–176 Sm^R^ (*C. jejuni* 81–176 *rpsL*^Sm^; DRH212) was electroporated with pABT739 and pDRH3260 to replace wild-type *Cjj81176_0266* or *Cjj81176_1237* with mutant alleles containing the *cat-rpsL* cassette. Transformants were recovered on MH agar containing chloramphenicol and verified by colony PCR to obtain ABT749 (81–176 *rpsL*^Sm^ *Cjj81176_0266*::*cat-rpsL*) and DRH3268 (81–176 *rpsL*^Sm^ *Cjj81176_1237*::*cat-rpsL*). ABT749 and DRH3268 were then electroporated with pABT745 and pABT656, respectively, to replace the *cat-rpsL* interrupted alleles with the in-frame deletion constructs on the chromosome. Transformants were recovered on MH agar with 0.5, 1, 2 or 5 mg ml^−1^ streptomycin and then screened for chloramphenicol sensitivity. Deletion of each gene was verified by colony PCR, which resulted in creation of ABT929 (81–176 *rpsL*^Sm^ Δ*Cjj81176_0266*) and ABT736 (81–176 *rpsL*^Sm^ Δ*Cjj81176_1237*).

Generation of a *C. jejuni* 81–176 mutant lacking genes for all putative hemerythrins was accomplished first by electroporating pABT739 into ABT477 (81–176 *rpsL*^Sm^ Δ*fedA*) to result in ABT757 (81–176 *rpsL*^Sm^ Δ*fedA Cjj81176_0266*::*cat-rpsL*) (Barrero-Tobon and Hendrixson, 2012). This strain was then electroporated with pABT745 to generate ABT780 (*C. jejuni* 81–176 *rpsL*^Sm^ Δ*fedA* Δ*Cjj81176_0266*). ABT780 was then electroporated with pDRH3260 to result in the creation of ABT835 (81–176 *rpsL*^Sm^ Δ*fedA* Δ*Cjj81176_0266 Cjj81176_1237*::*cat-rspL*). This strain was subsequently electroporated with pABT656 to generate the ABT867 (81–176 *rpsL*^Sm^ Δ*fedA* Δ*Cjj81176_0266* Δ*Cjj81176_1237*), which lacks genes for all three putative hemerythrins. Construction of all strains was verified by colony PCR.

### RNA extraction and RT-PCR

Overnight starter cultures of NCTC 11168 and *cj0241*::*kan* were used to inoculate MHS media, equilibrated under microaerobic conditions, to an OD_600_ of 0.1. Once the cultures had grown to mid-exponential phase (OD_600_ ∼0.4), a 10 ml aliquot was added to a pre-cooled RNase-free tube containing 32.5 μl phenol (Sigma) and 1.2 ml chilled ethanol. The sample was agitated briefly by vortex before centrifugation (4°C, 9200 r.p.m., 5 min). Bacterial pellets were stored at − 80°C. Extraction of cellular mRNA was performed using an adapted Tri Reagent method (Chomczynski, 1993) and, following treatment with TURBO DNase (Applied Biosystems), was quantified using a Nanodrop spectrophotometer (Thermo Scientific). For non-quantitative RT-PCR, cDNA was synthesized from extracted mRNA using SuperScript III Reverse Transcriptase (Invitrogen) as per the manufacturer's instructions, and PCRs were performed with primers (Table 1) designed to anneal to sequences within the coding region of *cj0241c*, *cj0239c* and *iscS*. The products were analysed by agarose gel electrophoresis. Control reactions without added RT produced no PCR products.

For qRT-PCR, RNA extracted from cultures of NCTC 11168 and *cj0241c*::*kan* before and after 4.5 h aerobic incubation was used to generate cDNA as above. Primers for qRT-PCR are listed in Table 1. Reactions were carried out in 96-well plates and prepared using the SensiMix SYBR Low-ROX kit (Bioline) according to manufacturer's instructions. Each 25 μl reaction contained 12.5 μl SensiMix SYBR Low-ROX buffer, 250 nM of each primer and 50 ng template. Plates were designed to include three technical and biological replicates of both a tested gene and the constitutively expressed internal control gene *rpoA* (Ritz *et al*., 2009), as well as a genomic DNA standard curve for each gene. Reactions were carried out using a MX3005P thermal cycler (Stratagene, La Jolla, CA, USA) with the following conditions: 1 cycle of 95°C for 10 min; 40 cycles of 95°C for 30 s, 55°C for 60 s (during which SYBR Green fluorescence was measured), 72°C for 60 s; and 1 cycle of 95°C for 60 s, 55°C for 30 s, 95°C for 30 s. Data were analysed and relative levels of expression calculated as previously described (Guccione *et al*., 2010).

### Overproduction and purification of hemerythrins

Overproduction of HerA with a C-terminal his-tag was achieved by PCR amplification of the *cj0241c* gene using primers 0241_OE F and 0241_OE R, and cloning the 422 bp product into pET-21a(+) (Merck Chemicals, Hoddesdon, UK) creating the plasmid pET0241. *E. coli* BL-21 (DE3) (pET0241) was grown at 37°C in LB medium containing 50 μg ml^−1^ carbenicillin to OD_600_ 0.6 before overexpression of *cj0241c* was induced by addition of 1 mM isopropyl-β-D-thiogalactopyranoside. Induced cultures were incubated overnight at 25°C with shaking at 250 r.p.m. before harvesting the cells by centrifugation (8800 r.p.m., 10 min, 4°C). Cell pellets were resuspended in binding buffer [20 mM phosphate buffer pH 7.4, 0.5 mM NaCl, 20 mM imidazole (Sigma)] before disruption by sonication (MSE Soniprep, 6 × 15 s bursts). Filter-sterilized CFE containing the His-tagged HerA protein was injected onto a 5 ml HisTrap HP column (GE Healthcare, Little Chalfont, UK) and eluted by a 0.02–0.5 M imidazole gradient. The HerA protein was eluted at ∼0.26 M imidazole. Eluted protein was concentrated to 1.5 mM using a Vivaspin 20 spin column (Sartorius Stedim, Epsom, UK) before resonance Raman spectroscopy. Protein was stored at 4°C. The hemerythrins Cj0045 (NCTC 11168), HerB (NCTC 11168) and FedA (81–176) were overproduced in the same vector system and using the same induction and purification conditions, using the primers listed in Table 1.

### Resonance Raman spectroscopy

Raman spectra were recorded on a home-built system. The 514.5 nm laser line of a Coherent Innova 300 CW Ar ion laser (Coherent Inc, Santa Clara, CA, USA) was used as the excitation source. The excitation power was maintained at 200 mW. The detection system was comprised of a Bentham TMC600 spectrograph (Bentham Ltd, Reading, UK) (1200 gr/mm grating employed) coupled with an Andor iDus DU440A CCD camera (Andor, Belfast, UK). To suppress the Rayleigh scattering, a razor-edge optical filter (LP03-532RU; Semrock, Rochester, NY, USA) was set in front of the entrance slit of the spectrograph, tilted relative to the beam axis to match the excitation wavelength. Right-angle detection arrangement was employed. The excitation light was focused into the sample cell in the vertical direction with a short-focus lens (ca. 15 mm) in the immediate proximity to the front wall of the cell. The sample (1.5 mM protein concentration) was placed into a customized 2 mm path-length quartz cell (Starna Scientific, Ilford, UK) with polished side-walls to allow access of the excitation beam.

## Supporting Information

Fig S1 Protein sequence alignments of hemerythrins in *C*. *jejuni* NCTC 11168 (top line), *C. jejuni* 81116 (middle line) and *C. jejuni* 81–176 (bottom line). HerA = Cj0241, HerB = Cj1224 and FedA = Cj0045 in strain NCTC 11168. Conserved motifs characteristic of hemerythrin proteins are highlighted in yellow, hydrophobic residues potentially lining the oxygen binding pocket are in blue and underlined (French *et al*., 2008). Potential metal binding CXC and CXXC motifs are highlighted grey. Amino acid similarity is indicated underneath the alignment: * = fully conserved residue: = residues sharing strongly similar properties. = residues sharing weakly similar properties. Sequence length is indicated above each protein; / = 20 residues. *FedA** indicates the missing C-terminal sequence of the 81–176 gene is encoded in a different reading frame. **Fig. S2.** *In vivo* recovery of Por and Oor activity after partial (A, B) or complete (C, D) aerobic inactivation. Assays of Por (A, C) and Oor (B, D) activity in CFE prepared from wild-type (closed circles) and *herA* mutant (open circles) cells incubated aerobically (500 ml cultures in 2.5 L baffled flasks with 250 r.p.m. shaking in air) for 4.5 h (A, B) or 16 h (C, D), followed by anaerobic incubation in the presence of chloramphenicol. Dashed vertical lines indicate the point of transfer from aerobic conditions to the anaerobic recovery conditions. All activities are expressed as a percentage of the initial pre-stress activity (mean initial values in μmol min − 1 mg protein − 1 A: wt = 2.4, *herA* = 2.3; B: wt = 0.61, *herA* = 0.61; C: wt = 1.0, *herA* = 0.9; D: wt = 0.18, *herA* = 0.17). The data shown in A and B are from four independent experiments, with error bars showing standard deviation. The data shown in C and D are from a single experiment which was repeated twice with similar results. **Fig. S3.** A. *Inset*: 12% SDS-PAGE gel showing purified NCTC 11168 HerB (Cj1224) protein band of ∼23 kDa (green arrow). Sizes of marker bands are also shown. *Main figure*: Absorption spectra of the purified as-isolated HerB (green spectrum) and HerB reduced with sodium dithionite (blue spectrum). B. Absorption spectra of purified as-isolated HerB (green spectrum) and HerB plus 50 mM sodium azide (orange spectrum). C. *Inset*: 12% SDS-PAGE gel showing purified 81–176 FedA protein band (green arrow). *Main figure*: Absorption spectra of as-isolated FedA purified from cells grown in media supplemented with ferrous ammonium sulphate (green spectrum), FedA reduced with sodium dithionite (blue spectrum) and FedA purified from cells grown with no additional ferrous ions in the medium (grey line). D. UV absorption spectra of purified iron-loaded FedA (green spectrum) and FedA with 50 mM sodium azide (orange spectrum). **Table S1.** Comparison between the components of the *E. coli* and *C. jejuni* NCTC 11168 Fe-S cluster biosynthesis pathways. Protein function is listed along the top row, and the specific pathways that the listed proteins belong to are in the right-hand column (other referring to those proteins not experimentally linked to any specific pathway) (Py and Barras, 2010). *Campylobacter jejuni* homologues to *E. coli* proteins are shown in bold with percentage sequence identity in brackets. Note that Cj0239 is homologous with the characterized NifU protein in *H. pylori* (Hp) rather than IscU, so this comparison is also shown. Proteins without homologues in *C. jejuni* are indicated by a − sign.
