# Supplementary material for: Hemerythrins in the microaerophilic bacterium Campylobacter jejuni help protect key iron–sulphur cluster enzymes from oxidative damage
Source: Environ Microbiol. 2013 Dec 17;16(4):1105–21. doi: 10.1111/1462-2920.12341 (PMC4257069; doi:10.1111/1462-2920.12341)
Supplement: Fig S1 — Protein sequence alignments of hemerythrins in C. jejuni NCTC 11168 (top line), C. jejuni 81116 (middle line) and C. jejuni 81–176 (bottom line). HerA = Cj0241, HerB = Cj1224 and FedA = Cj0045 in strain NCTC 11168. Conserved motifs characteristic of hemerythrin proteins are highlighted in yellow, hydrophobic residues potentially lining the oxygen binding pocket are in blue and underlined (French et al., 2008). Potential metal binding CXC and CXXC motifs are highlighted grey. Amino acid similarity is indicated underneath the alignment: * = fully conserved residue: = residues sharing strongly similar properties. = residues sharing weakly similar properties. Sequence length is indicated above each protein; / = 20 residues. FedA* indicates the missing C-terminal sequence of the 81–176 gene is encoded in a different reading frame. Fig. S2. In vivo recovery of Por and Oor activity after partial (A, B) or complete (C, D) aerobic inactivation. Assays of Por (A, C) and Oor (B, D) activity in CFE prepared from wild-type (closed circles) and herA mutant (open circles) cells incubated aerobically (500 ml cultures in 2.5 L baffled flasks with 250 r.p.m. shaking in air) for 4.5 h (A, B) or 16 h (C, D), followed by anaerobic incubation in the presence of chloramphenicol. Dashed vertical lines indicate the point of transfer from aerobic conditions to the anaerobic recovery conditions. All activities are expressed as a percentage of the initial pre-stress activity (mean initial values in μmol min − 1 mg protein − 1 A: wt = 2.4, herA = 2.3; B: wt = 0.61, herA = 0.61; C: wt = 1.0, herA = 0.9; D: wt = 0.18, herA = 0.17). The data shown in A and B are from four independent experiments, with error bars showing standard deviation. The data shown in C and D are from a single experiment which was repeated twice with similar results. Fig. S3. A. Inset: 12% SDS-PAGE gel showing purified NCTC 11168 HerB (Cj1224) protein band of ∼23 kDa (green arrow). Sizes of marker bands are also shown. Main [file emi0016-1105-SD1.pdf]

	Cysteine desulphurase		Scaffold		ATP-hydrolysing component		Trafficking	
	E. coli	*C. jejuni* NCTC 11168	*E. coli/ H.pylori*	*C. jejuni* NCTC 11168	*E. coli*	*C. jejuni* NCTC 11168	*E. coli*	*C. jejuni* NCTC 11168
ISC/NIF	IscS	Cj0240 (39%)	IscU (*Ec*)<br>NifU (*Hp*)	Cj0239 (40%)<br>Cj0239 (69%)	HscA<br>HscB	DnaK (41%)<br>-	IscA	-
SUF	SufS<br>SufE	Cj0240 (27%)<br>-	SufB<br>SufD	-<br>-	SufC	LivG (26%)<br>Cj0669 (28%)<br>Cj1663 (27%)	SufA	-
Other	CsdA	Cj0240 (27%)<br>Cj0791 (22%)					Mrp<br>NfuA<br>ErpA	Cj1606 (37%)<br>Cj1639 (32%)<br>-

Supplementary Table S1. Comparison between the components of the *E. coli* and *C. jejuni* NCTC 11168 Fe-S cluster biosynthesis pathways. Protein function is listed along the top row, and the specific pathways that the listed proteins belong to are in the right-hand column (other referring to those proteins not experimentally linked to any specific pathway) (Py & Barras, 2010). *C. jejuni* homologues to *E. coli* proteins are shown in bold with percentage sequence identity in brackets. Note that Cj0239 is homologous with the characterised NifU protein in *H. pylori* (Hp) rather than IscU, so this comparison is also shown. Proteins without homologues in *C. jejuni* are indicated by a − sign.

```
HerA                          /                        /                   /                   /                   /
11168     -MTYNEKIISMNNDLLDHQHKELFEIS-----KKLSLMNQRHVGTKELKIVLRELLIMINRHFSDEEAFMREIEYPYINHHTRIHRKIILEIEEIIISEAKFVNIMTEKLNLVVQDFIFK...
81116     -MTYNEKIISMNNDLLDHQHKELFEIS-----KKLSLMNQRHVGTKELKIVLRELLIMINRHFSDEEAFMREIEYPYINHHTRIHRKIILEIEEIIISEAKFVNIMTEKLNLVVQDFIFK...
81-176    -MTYNEKIISMNNDLLDHQHKELFEIS-----KKLSLMNQRHVGTKELKIVLRELLIMINRHFSDEEAFMREIGYPYINHHTRIHRKIILEIEEIIISEAKFVNIMTEKLNLVVQDFIFK...
           **************************     ***************************************** **********************************************
HerB                         /                        /                   /                   /                   /
11168     MLPKWDNSYSVHNAKIDEQHKKLFKLA-----AKVEVVSDRSVSKNEVKELLAEFFNYMKDHFNDEEKYMQLIGYPNLEEHRKIHKEIIQTMINLIK-DIKSTNDLKEKLYIVAKKWLLE...
81116     MLPKWDNSYSVHNAKIDEQHKKLFELA-----AKVEVVSDRSVSKNEVKELLAEFFNYMKDHFNDEEKYMQLIGYPNLEEHRKIHKEIIQTMINLIK-DIKSTNDLKEKLYIVAKKWLLE...
81-176    MLPKWDNSYSVHNAKIDEQHKKLFKLA-----AKVEVVSDRSVSKNEVKELLAEFFNYMKDHFNDEEKYMQLIGYPNLEEHRKIHKEIIQTMINLIK-DIKSTNDLKEKLYIVAKKWLLE...
          ************************:**     ***************************************************************** **********************
FedA                         /                   /                   /                   /                   /
11168     MKVKWSRDFSIKNMQLDKQHELIFEITNLANDLALNIQDNNTQHKNDLKQILVKLFQYIKIHFKDEEKFMESIDFPLIEEHKKSHQILVEKTKELLE-HSNDIVKMSQELSILTKDWILD...
81116     MKVKWSRDFSIKNMQLDKQHELIFEITNLANDLALKIQENNTQYKDDLKQILAKLFQYIKIHFKDEEKFMESIDFPLIEEHKKSHQILVEKTKELLE-HSDNIVKMSQELSILTKDWILD...
81-176    MEVKWSRDFSIKNMQLDKQHELIFEITNLANDLALNIQDNNTQHKNDLKQILVKLFQYIKIHFKDEEKFMESIDFPLIEEHKKSHQILVEKTKELLE-HSNDIVKMSQELSILTKDWILD...
          *:*********************************:**:****:*:******:******************************************** **::******************

HerA              /
11168     ...HTAKEDSKIVKYYEEKFKK 133
81116     ...HTAKEDSKIVKYYEEKFKK 133
81-17     ...HTAKEDSKIVKYYEEKFKK 133
             *******************
HerB              /                   /                       /                   /
11168     ...HILYEDMKVEKWRSSSLSTDDGGDVSFEAAEDE----DNEHPQFYLYTCNCPGKIHDVPYSIHQKIELQGRKFTCKTCKQAIKFYKKYS 199
81116     ...HILYEDMKVEKWRSSSLSTDDGGDVSFEAAEDE----DNEHPQFYLYTCNCPGKIHDVPYSIHQKIELQGHKFTCKTCKQAIKFYKKHS 199
81-176    ...HILYEDMKVEKWRSSSLSTDDGGDISFEAAEDE----DNEHPQFYLYTCNCPGKIHDVPYSIHQKIELQGHKFTCKTCKQAIKFYKKYS 199
             ************************:********    *********************************:****************:*
FedA         /                   /                   /                   /                   /                   /                   /
11168     ...HFANEDLWIANFTKKTLHLQEIHYTLEQYIKLKSIKQDLRAEKTYDYICNCSLRIHAVPQTIHQELVSKENTLKCEKCGQILVHLDYFDLNQNFEKFNAIFEDALQNHHFTTQENDMRGGG 240
81116     ...HFANEDLWIANFTKKTLHLQEIHYTLEQYIKLKSIKQDLRAEKTHDYICNCSLRIHAVPQTIHQELVSKENTLKCEKCGQILVHLDYFDLNQNFEKFNAIFEDALQNHHFTTQKNDMGGG- 239
81-176    ...HFANEDLWIANFTKKP-------YTFKKSIIP----------------------------------------------------------------------------------------- 145
FedA*        --------------------------------KSIKQDLRAEKTHDYICNCYLRTHAVPQTIHEELVSKENTLKCEKCGQILVHLDYFDLNQNFEKFNAIFEDALQNHHFTTQKNDMGGG- 88
             ***************.       **::: *  ************:****** ** ********:*************************************************:*** **
```

Supplementary Figure 1: Protein sequence alignments of hemerythrins in *C. jejuni* NCTC 11168 (top line), *C. jejuni* 81116 (middle line) and *C. jejuni* 81-176 (bottom line). HerA = Cj0241, HerB = Cj1224 and FedA = Cj0045 in strain NCTC 11168. Conserved motifs characteristic of hemerythrin proteins are highlighted in yellow, hydrophobic residues potentially lining the oxygen binding pocket are in blue and underlined (French *et al.*, 2008). Potential metal binding CXC and CXXC motifs are highlighted grey. Amino-acid similarity is indicated underneath the alignment: * = fully conserved residue, : = residues sharing strongly similar properties, . = residues sharing weakly similar properties. Sequence length is indicated above each protein; / = 20 residues. *FedA** indicates the missing C-terminal sequence of the 81-176 gene is encoded in a different reading frame.

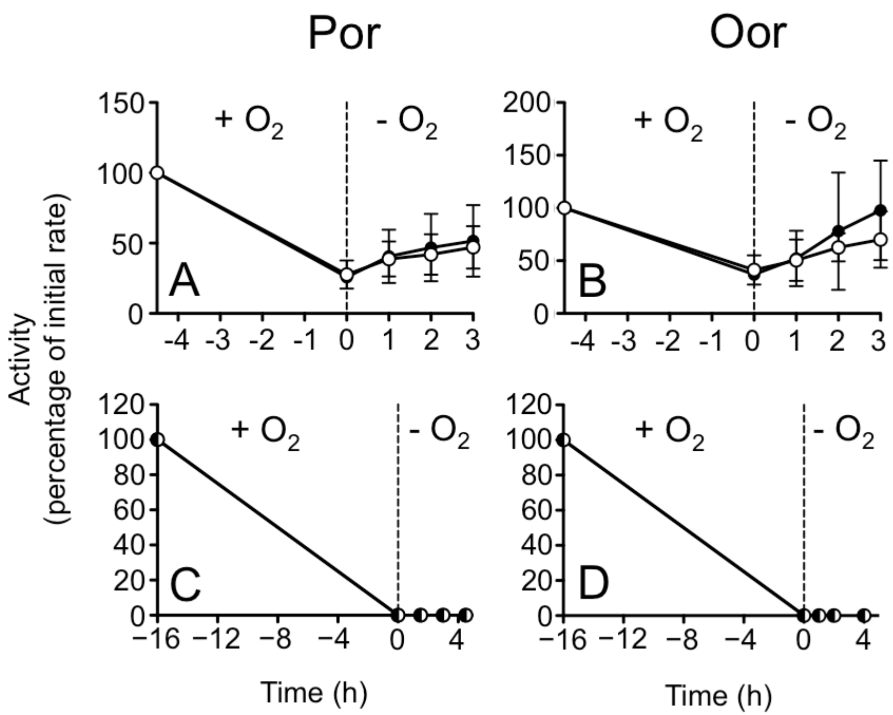

Supplementary Figure 2. *In vivo* recovery of Por and Oor activity after partial (A, B) or complete (C, D) aerobic inactivation. Assays of Por (A, C) and Oor (B, D) activity in CFE prepared from wild-type (closed circles) and *herA* mutant (open circles) cells incubated aerobically (500 ml cultures in 2.5 L baffled flasks with 250 rpm shaking in air) for 4.5 hours (A, B) or 16 hours (C, D), followed by anaerobic incubation in the presence of chloramphenicol. Dashed vertical lines indicate the point of transfer from aerobic conditions to the anaerobic recovery conditions. All activities are expressed as a percentage of the initial pre-stress activity (mean intial values in μmol min^{-1} mg $protein^{-1}$ A: wt = 2.4, *herA* = 2.3; B: wt = 0.61, *herA* = 0.61; C: wt = 1.0, *herA* = 0.9; D: wt = 0.18, *herA* = 0.17). The data shown in A and B are from four independent experiments, with error bars showing standard deviation. The data shown in C and D is from a single experiment which was repeated twice with similar results.

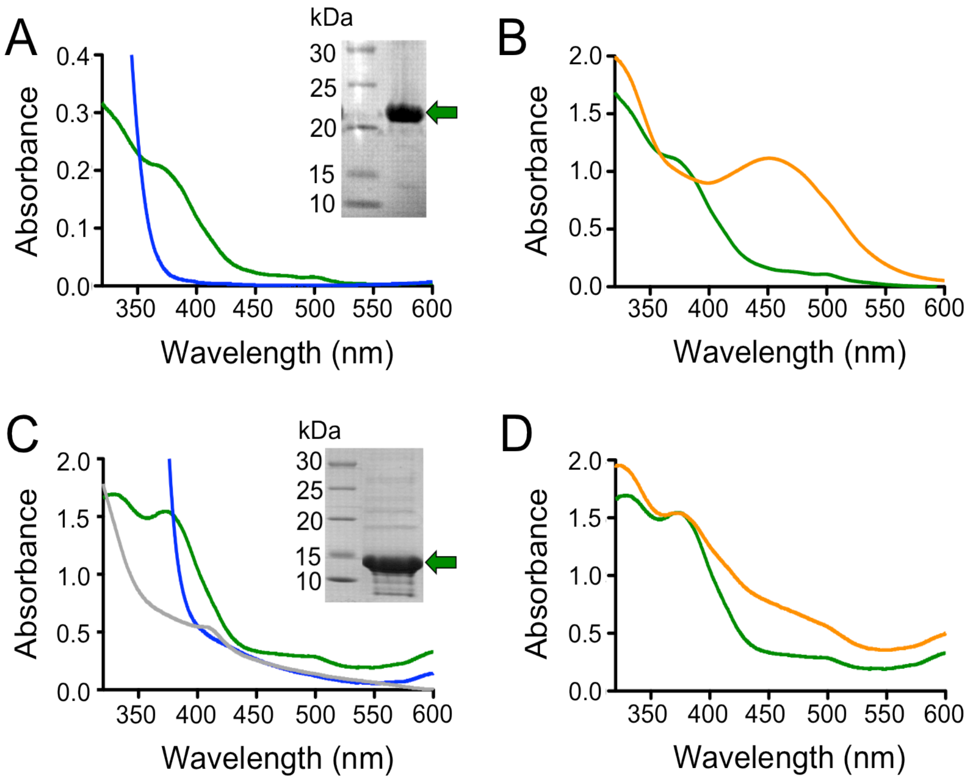

Supplementary Figure 3. (**A**) *Inset*: 12% SDS-PAGE gel showing purified NCTC 11168 HerB (Cj1224) protein band of ~23 kDa (green arrow). Sizes of marker bands are also shown. *Main figure*: Absorption spectra of the purified as-isolated HerB (green spectrum) and HerB reduced with sodium dithionite (blue spectrum). (**B**) Absorption spectra of purified as-isolated HerB (green spectrum) and HerB plus 50 mM sodium azide (orange spectrum). (**C**) *Inset*: 12% SDS-PAGE gel showing purified 81-176 FedA protein band (green arrow). *Main figure*: Absorption spectra of as-isolated FedA purified from cells grown in media supplemented with ferrous ammonium sulphate (green spectrum), FedA reduced with sodium dithionite (blue spectrum) and FedA purified from cells grown with no additional ferrous ions in the medium (grey line). (**D**) UV absorption spectra of purified iron-loaded FedA (green spectrum) and FedA with 50 mM sodium azide (orange spectrum).
